# CAR‐T Cell Therapy for PTLD: Analysis of CAR‐T Product, Engraftment, and Outcomes in Patients Receiving Parallel Immunosuppression

**DOI:** 10.1002/jha2.70006

**Published:** 2025-02-20

**Authors:** Aikaterini Panopoulou, Vedika Mehra, Kate Cwynarski, Andrew Morley‐Smith, Angela Hwang, Maeve O'Reilly, Harriet Roddy, Claire Roddie

**Affiliations:** ^1^ University College London Hospital NHS Foundation Trust London UK; ^2^ University College London Cancer Institute London UK; ^3^ Harefield Hospital Guy's & St Thomas’ NHS Foundation Trust London UK

**Keywords:** CAR‐T, corticosteroids, PTLD, SOT, tacrolimus, T‐cell fitness

1

Post‐transplant lymphoproliferative disorder (PTLD) is a potentially life‐threatening complication of solid organ transplantation (SOT) [1]. Following FDA approval in large B‐cell lymphoma (LBCL), PTLD patients can now access CD19‐directed chimeric antigen receptor T‐cell (CAR‐T) therapy, but having been excluded from pivotal trials, there are limited clinical data on response/toxicity and peri‐CAR‐T immunosuppression (IS) management. Lifelong IS is critical to prevent organ rejection but may be detrimental to CAR‐T function/expansion [2] which may adversely impact clinical responses [3, 4].

Here, we describe third‐line CD19CAR‐T therapy with axicabtagene ciloleucel (axi‐cel) in two PTLD patients who continued therapeutic IS throughout to protect graft function. In parallel, we performed CAR‐T product and peripheral blood (PB) CAR‐T marking analysis. Clinical and laboratory methods are detailed in Supporting Information appendix.

*Patient 1 (P1)*: A 24‐year‐old male had received orthotopic cardiac transplant at 3 years of age for epstein barr virus (EBV)‐related myocarditis and commenced lifelong tacrolimus/azathioprine. He developed EBV‐negative monomorphic PTLD and received five lines of therapy for multiple relapse events prior to axi‐cel referral (detailed in Table S1). Leukapheresis was performed without tacrolimus interruption. PB lymphocyte count was 0.72 × 10^9^/L, CD3+ count was 0.68 × 10^9^/L, and total harvested CD3+ yield was 2.64 × 10^9^. Despite therapeutic tacrolimus, the product fulfilled manufacturing release criteria. Bridging comprised rituximab, bendamustine, and polatuzumab vedotin (RBP) to progressive disease (PD).

He received fludarabine/cyclophosphamide/CAR‐T infusion and developed grade (G)1 CRS on day 1 (but no ICANS/graft rejection), receiving tocilizumab 8 mg/kg. Tacrolimus remained within the 5–7 ng/mL target range. Day 28 PET‐CT showed complete metabolic response (CMR). Month 3 PET‐CT showed a single avid para‐aortic node, treated with 40 Gy radiotherapy to CMR, ongoing at month 12 (Figure S1a).

*Patient 2 (P2)*: A 51‐year‐old male underwent cadaveric renal transplantation for polycystic kidney disease. He developed EBV+ monomorphic PTLD 2 months post‐SOT which was refractory to six lines of treatment (detailed in Table S1). His latest line of therapy was one cycle of RBP, delivered 32 days prior to apheresis. IS comprised prednisolone monotherapy (20 mg/day). Pre‐leukapheresis, his PB lymphocyte and CD3+ T‐cell counts were 0.24 × 10^9^/L and 0.11 × 10^9^/L. His lymphocyte count had been consistently ≤0.3 × 10^9^/L for >1 year after steroids/prior therapies, and the total CD3+ T‐cell yield from leukapheresis was 1.39 × 10^9^. The resulting CAR‐T product was out‐of‐specification (OOS) due to low cell viability (71% against a specification of ≥80%) but was approved for infusion due to rapidly progressive disease. Bridging comprised RBP to PD, and 20 Gy radiotherapy to the oropharynx/thorax (Figure 1A). The patient received Flu/Cy/CAR‐T infusion and continued prednisolone (10 mg/day) with normal renal function throughout. He developed G1 CRS on day 3 and slurred speech on day 6, presumed ICANS, and received 6‐hourly dexamethasone 10 mg, but his neurological status continued to deteriorate. CSF analysis revealed clonal B‐cells and MRI brain/spine confirmed neurological involvement by PTLD (Figure S1B). Due to rapidly progressive, debilitating disease, the patient opted for palliative care and passed away on day 30.

**FIGURE 1:**
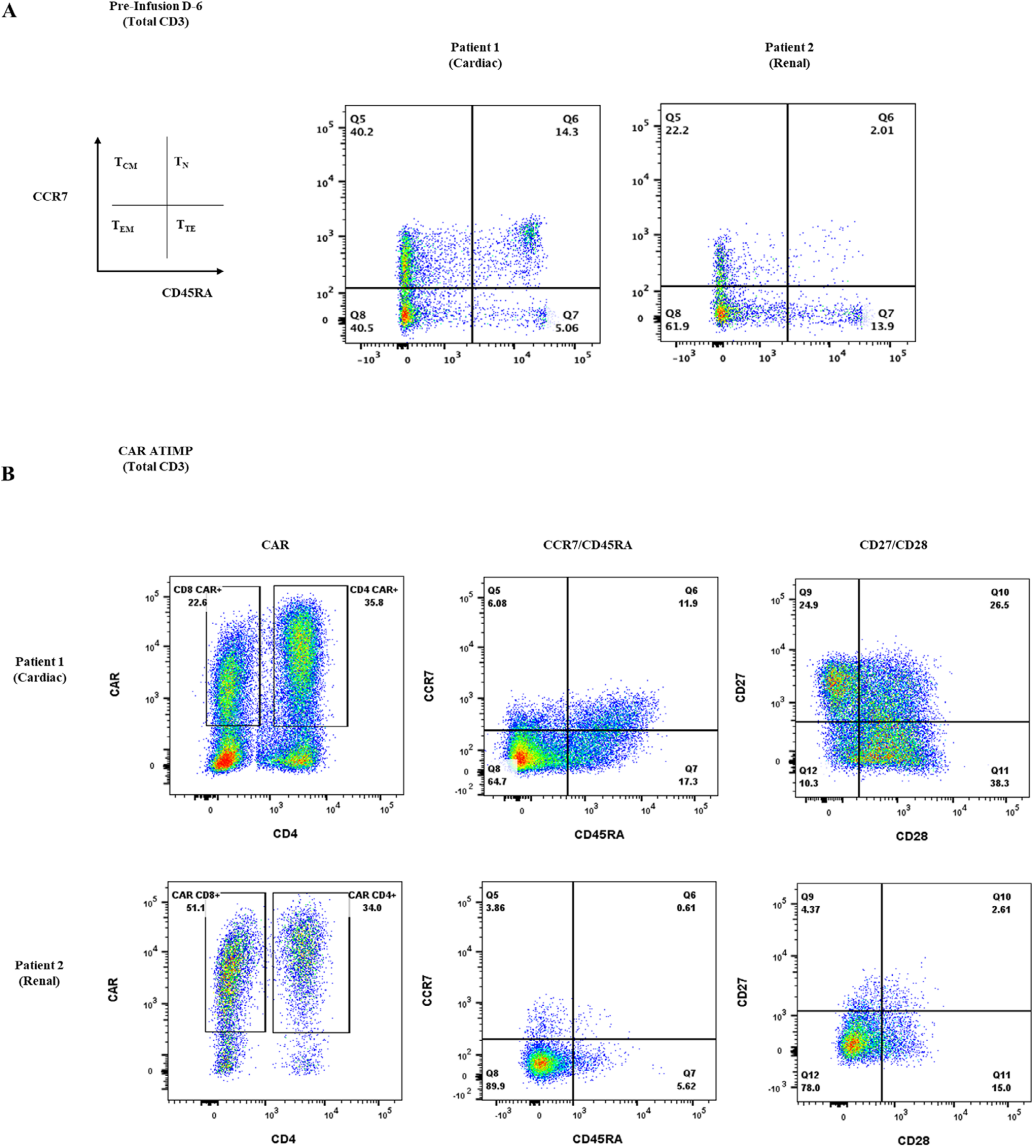
Characterization of baseline PB and CAR‐T product phenotype. (A) Flowcytometry plots depicting CCR7/CD45RA staining in baseline PB, 6 days prior to CAR‐T infusion. (B) Flowcytometry plots depicting CAR expression in CD4/8 T‐cell subsets, CCR7/CD45RA staining in total CAR+ populations and CD27/CD28 staining in total CAR+ populations in ATIMP CAR products. ATIMP = advanced therapy investigational medicinal product; PB = peripheral blood.

*Longitudinal PB lymphocyte subsets*: Table S2 illustrates pre‐leukapheresis, pre‐Flu/Cy, and follow‐up PB lymphocyte subsets. CD19+ B‐cell numbers were low throughout for both patients. Pre‐leukapheresis CD3 counts are demonstrably lower in P2 than P1, but low in both patients following bridging (Table S2). Overall, naïve T‐cell (Tn) populations were substantially lower than in healthy donor controls (Table S3A, Figure 1A), and P2 demonstrates enrichment for CD4+CD25+ regulatory T‐cells (Tregs), albeit overall T‐cell numbers were low (Table S3A). The overall picture likely reflects ongoing IS and prior lymphotoxic treatment (five and six lines, respectively), magnified in P2 by steroids and recent Bendamustine [5].

*CAR‐T product phenotype*: CAR expression was 58.4% and 85% for P1 and P2, respectively. CAR populations were predominantly effector/terminally differentiated and enriched for senescent phenotypes (CD27‐/CD28‐), more pronounced in P2 (Figure S1B; Table S3B). We were unable to run CAR product Treg analysis due to pauci‐cellular samples.

*CAR‐T expansion/persistence*: P1 demonstrates prompt CAR‐T engraftment and expansion up to day 28 despite ongoing therapeutic tacrolimus. By contrast, P2 shows poor CAR‐T engraftment and expansion (Figure 2A). Peak CAR‐T levels (cMax) and expansion (AUC^0‐28^) by qPCR are substantially lower in P2 than P1 (2610 vs. 10,814 and 30,232 vs. 257,233 copies/µg gDNA) (Figure 2B).

**FIGURE 2:**
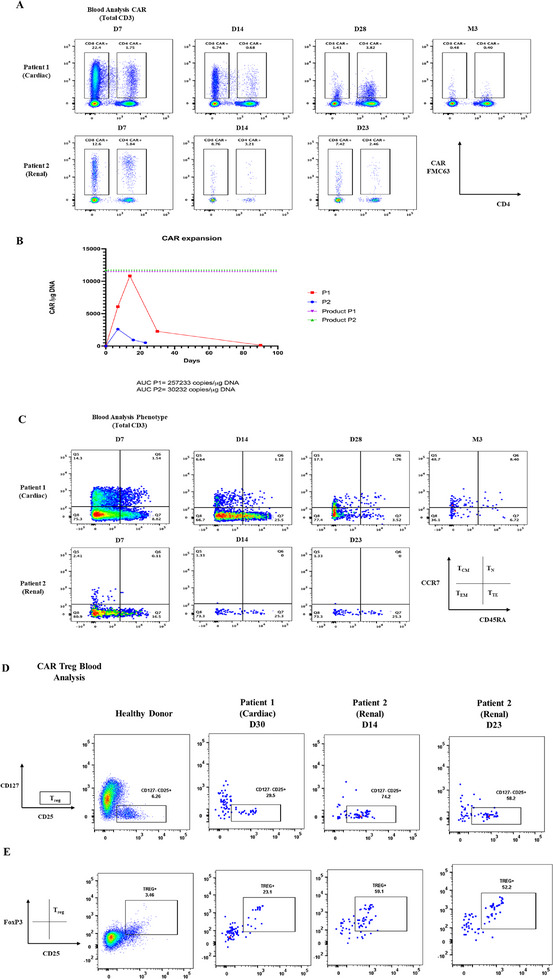
Longitudinal characterization of CAR‐T expansion, persistence, and CAR‐Tregs in PB. (A) Flowcytometry plots depicting PB CAR expression in CD4/8 T‐cell subsets at Day 7, 14, 23/28, and Month 3 timepoints post CAR‐T infusion. (B) CAR T cell expansion and persistence in PB, assessed by CAR‐specific qPCR at Day 7, 14, 23/28, and Month 3 timepoints post CAR‐T infusion. (C) Flowcytometry plots depicting CCR7/CD45RA staining in total CAR+ populations at Day 7, 14, 23/28, and Month 3 timepoints post CAR‐T infusion. (D) Flowcytometry plots depicting CD127/CD25 and (E) FoxP3/CD25 staining in total CAR+ populations to identify CAR‐Tregs at Day 28 for patient 1 and Days 14/23 for patient 2. Expression form total CD4 T‐cells stained in a healthy donor were used as a staining/gating control. PB = peripheral blood.

Longitudinal PB CAR‐T phenotyping in P1 illustrates circulating naïve/central memory (Tn/Tcm) CAR‐T subsets, whereas terminally differentiated (Te/Tte) and senescent populations (CD27‐/CD28‐) predominate in P2 (Figure 2C; Table S4). Extended Treg stains (CAR+/CD4+/CD25+/CD127‐/FOXP3+) demonstrate enrichment for CAR‐Tregs in P2 (Figure 2E).

To date, clinical results of CAR‐T therapy for PTLD give room for cautious optimism [6, 7, 8]. One retrospective analysis of 22 patients [9] demonstrates CR in 55%, with 2‐year PFS and OS of 35% and 58%, not dissimilar to outcomes for non‐PTLD LBCL. In our analysis, we show durable CR in one patient and PD in the other patient.

Data suggest that immunotoxicity rates in PTLD are similar to non‐PTLD, but with higher NRM (∼9%–11%), driven predominantly by infection and encephalopathy [8, 9, 10]. SOT rejection affects ∼10%–25% of infused patients (majority renal) and is associated with IS interruption [6, 9]. The use of Flu/Cy and CAR‐induced hypogammaglobulinemia may help protect against early graft rejection. To date, cardiac SOT rejection has not been described post‐CAR‐T [9, 10, 11]. In our analysis, we continued IS throughout and saw minimal immunotoxicity and no rejection. Careful management of peri‐CAR‐T IS a key safety consideration.

The effect of therapeutic IS on CAR‐T manufacturing success and phenotype/expansion/persistence in vivo is sparsely reported in the literature [12, 13]. In our analysis, P2 had extremely low CD3+ counts pre‐harvest, likely from ongoing IS with steroids and prior lymphotoxins including bendamustine. It has been described that recent bendamustine exposure prior to apheresis (<6–9 months) can lead to poorer response and survival outcomes [5] and an increased risk of manufacture failure [14]. His CAR‐T product was OOS. CAR‐T product phenotyping for both patients revealed less desirable late effector/senescent populations [4], but P1 showed excellent early CAR‐T expansion (skewed toward CD8) despite ongoing tacrolimus. CAR‐T expansion was substantially lower in P2, likely due to product factors/steroids and prior lymphotoxins. While it is not clear that CAR Treg enrichment in P2 versus P1 was the cause for poor expansion, other studies have shown that CAR‐Tregs are associated with LBCL progression [15, 16].

We conclude that CAR‐T can be effective in heavily pretreated PTLD patients despite therapeutic IS. However, the cumulative impact of IS, low pre‐leukapheresis CD3+ counts, and prior lymphotoxins on CAR‐T products warrants further attention, and earlier referral may be key to improving outcomes.

## Author Contributions

C.R. designed the project, and V.M. and H.R. designed and performed the laboratory work. A.P., K.C., A.M., A.H., and M.O.R. compiled the clinical data. A.K., V.M., and C.R. wrote the manuscript. All authors edited and reviewed the manuscript.

## Conflicts of Interest

C.R. received honoraria from Kite Gilead, Novartis, and Bristol Myers Squibb. M.O.R. has served on advisory boards and received honoraria from Kite/Gilead, Novartis, and Janssen. K.C. has served on advisory boards and received honoraria from Kite Gilead, Bristol Myers Squibb, Abbvie, Roche, Takeda, Atara and Janssen. The remaining authors declare no conflicts of interest.

## Clinical Trial Registration

The authors have confirmed clinical trial registration is not needed for this submission.

## Supporting information



Supporting Information

## Data Availability

De‐identified data that support the findings of this study are available on request from the corresponding author.
