# Supplementary material for: CAR‐T Cell Therapy for PTLD: Analysis of CAR‐T Product, Engraftment, and Outcomes in Patients Receiving Parallel Immunosuppression
Source: EJHaem. 2025 Feb 20;6(1):e70006. doi: 10.1002/jha2.70006 (PMC11840709; doi:10.1002/jha2.70006)
Supplement: Supplementary file 1 — Supporting Information [file JHA2-6-e70006-s001.docx]

**SUPPLEMENTARY APPENDIX:**

**TITLE**

CAR-T cell therapy for PTLD: analysis of CAR-T product, engraftment, and outcomes in patients receiving parallel immunosuppression.

**Panopoulou et al.**

1. **SUPPLEMENTARY METHODS:**

***Clinical data***

Data was collected retrospectively from electronic medical records at University College London Hospital (UCLH) for patients treated with licenced CD19CAR-T (axicabtagene ciloleucel; axi-cel) for r/r PTLD at 3^rd^ line following authorisation from NHS England (NHSE). Data was processed according to declaration of Helsinki. PTLD was defined according to the WHO classification^1^. EBV status was defined by in situ hybridization for EBV encoded RNA or immunohistochemistry. Plasma EBV PCR was performed in a validated assay. Eligibility included age≥18 years; failure ≥2 lines of therapy; adequate organ function; ECOG PS 0-1. IS was continued through leukapheresis, bridging therapy (BT) and post infusion to preserve SOT function. Lymphodepletion (LD) comprising fludarabine and cyclophosphamide (Flu/Cy) was delivered as per Summary of Product Characteristics and local guidelines. CRS and Immune effector cell-associated neurotoxicity syndrome (ICANS) grading was performed as per American Society for Transplantation and Cellular Therapy (ASTCT) consensus^2^. Treatment response was assessed by fluorodeoxyglucose (FDG) positron emission tomography (PET-CT) pre-bridging therapy, pre-LD and at months 1 and 3 post-CAR-T^3^.

***Laboratory analysis***

T-cell ‘fitness’ was characterized phenotypically by the T-cell markers CD62L, CCR7, CD45RA, CD27 and CD28 which together define progressively more differentiated T-cell subsets on a continuum from naïve/stem-cell, to memory/central, to memory/effector to terminal subsets (Tn/ Tscm/ Tcm/ Te/ Tte)^4^. Assessment of PB immune cell subsets, manufactured axi-cel products and longitudinal CAR-T marking at days -6, 7, 14, 23, 28 and month 3 was performed on product bag wash or ammonium-chloride-potassium (ACK) lysed whole blood samples by flow cytometry on the BD LSRFortessa^TM^ and analysed using FlowJo v10 software. CD19 CAR FMC63 Idiotype Antibody, REAfinity^TM^ (REA1297) (Miltenyi Biotec) was used to detect CAR-T. Fixable viability dye eFluor™ 780 (Invitrogen^TM^), was used to discriminate live/dead cells. Antibodies used in this analysis include, anti-human CD4 (RPA-T4), CD8 (RPA-T8), CD45RA (HI100), CCR7 (G025H7), CD27 (M-T271), CD28 (CD28.2), CD25 (BC96) and CD127 (A019D5), all were obtained from BioLegend. Following surface staining, intracellular staining for FoxP3 (PCH101) (Invitrogen) was performed following fixation/permeabilization (BD Pharmingen Transcription-Factor Buffer Kit). DNA for CAR-T qPCR was extracted from the CAR-T product and from PB samples using Qiagen DNeasy® Blood and Tissue kit according to manufacturer’s instructions. Assessment of CAR-T marking was performed using a qPCR assay to detect viral copy number insertion by measurement of the viral packaging signal of the CAR-T construct in copies/µg gDNA. AUC(D0-28) was performed using GraphPad Prism software.

**SUPPLEMENTARY TABLES:**

**Sup. Table 1:** Summary of SOT, PTLD and treatment details. Key: L= line of therapy IS=Immunosuppression; BT= Bridging Therapy; CR=Complete Response, PD=Progressive Disease, MR=Mixed Response; R-COP=Rituximab, Cyclophosphamide, Vincristine, Prednisolone; R-COPADM=Rituximab, Cyclophosphamide, Vincristine, Prednisolone, Doxorubicin, Methotrexate; R-CYM= Rituximab, Cytarabine, Methotrexate; R-GDP= Rituximab, Gemcitabine, Dexamethasone, Cisplatin; R-IVE= Rituximab, Ifosphamide, Vincristine, Epirubicin; R-ICE= Rituximab, Ifosphamide, Carboplatin, Etoposide; R-CHOP= Rituximab, Cyclophosphamide, Doxorubicin, Vincristine, Prednisolone; R-Gem-Ox= Rituximab, Gemcitabine, Oxaliplatin, R-BP=Rituximab, Bendamustine, Polatuzumab.* No EBV-matched CTL products available to this patient as therapeutic option.

| **P#** | **SOT details** | | | **PTLD details** | | | | **Rx details** | | |
| --- | --- | --- | --- | --- | --- | --- | --- | --- | --- | --- |
|  | **Age** | **Graft source /type/ date** | **IS** | **Time from SOT (M)** | **Type DLBCL/**  **EBV status** | **Stage** | **LDH/ range/ at referral** | **Prior Rx for PTLD** | **BT** | **Disease Status at CAR-T** |
| P1 | 24 | Cadaveric, orthotopic, cardiac | Tac | 180 m/ 15y | DLBCL  EBV -ve | IVE | 128  (135-225 IU/L) | **1L:** azathioprine stopped and tacro dose reduction (PD)  **2L:** R-COP, 2x R-COPADM; 2x R-CYM (CR)  **3L:** 2x R-GDP (PD)  **4L:** 2x R-IVE (CR)  **5L:** 2x R-ICE (PD) | 1x RBP | PD |
| P2 | 51 | Cadaveric, heterotopic, renal | Pred | 2m | DLBCL  EBV +ve* | IVE |  | **1L:** Reduction IS (PD)  **2L:** Rituximab (PD)  **3L:** 3x R-CHOP (PD)  **4L:** Nanatinostat (PD)  **5L:** 2x R-Gem-Ox (PD)  **6L:** 1x R-BP (MR) | 1x RBP + 20Gy IFRT (oropharynx/ thorax) | PD |

**Sup. Table 2:** Summary of longitudinal immune cell subsets by flow cytometry.

|  | **Pre-leukapheresis** | | **Pre-LD** | | **M1** | **M3** |
| --- | --- | --- | --- | --- | --- | --- |
|  | **P1** | **P2** | **P1** | **P2** | **P1** | **P1** |
| **CD19 absolute**  *NR 0.11 - 0.69 x10^9/L* | 0.000 | 0.003 | 0.000 | 0.001 | 0.000 | 0.001 |
| **CD3 absolute**  *NR 0.87 - 2.51 x 10^9/L* | 0.68 | 0.11 | 0.29 | 0.10 | 0.30 | 0.18 |
| **CD4 absolute**  *NR 0.44 - 1.47 x 10^9/L* | 0.38 | 0.03 | 0.17 | 0.04 | 0.16 | 0.10 |
| **CD4:CD8 ratio**  *NR 0.54 - 2.97* | 1.47 | 0.36 | 1.78 | 0.62 | 1.36 | 1.23 |
| **CD8 absolute**  *NR 0.25 - 0.99 x 10^9/L* | 0.259 | 0.08 | 0.095 | 0.057 | 0.117 | 0.077 |

**Sup. Table 3:** Summary of baseline pre-infusion blood and CAR-T product immune cell subsets by flow cytometry. **(A)** Percentage CD4/CD8 subsets in baseline patient and healthy donor PB, gated from total live cells. Percentage Tn (CCR7+/CD45RA+), Tcm (CCR7+/CD45RA−), Te (CCR7−/CD45RA−) and Tte (CCR7−/CD45RA+), CD27+/CD28+ and CD25+ subsets are sub gated from respective CD4/8 populations.

| Baseline Blood Analysis (%) | Pre-Infusion Blood Analysis (%) | | Baseline Healthy Donor |
| --- | --- | --- | --- |
|  | **Patient 1**  **(Cardiac)** | **Patient 2 (Renal)** | **Healthy Donor (Mean ±SD, n=6)** |
| CD4 | 74.3 | 47.6 | 72.9 (± 7.5) |
| CD8 | 25.7 | 52.4 | 27.1 (± 7.6) |
| CD4 Tn | 1.7 | 1.4 | 40.5 (± 16.4) |
| CD4 Tcm | 49.8 | 39.3 | 24.7 (± 5.3) |
| CD4 Te | 48 | 58.7 | 33.9 (± 17.7) |
| CD4 Tte | 0.5 | 0.6 | 1 (± 0.5) |
| CD4 CD27+/CD28+ | 54.7 | 69.3 | 82.6 (± 13.7) |
| CD4 CD25+ | 11 | 31 | 8 (± 2.8) |
| CD8 Tn | 50.6 | 2.6 | 50.9 (± 15.2) |
| CD8 Tcm | 12.4 | 6.7 | 3.7 (± 1.4) |
| CD8 Te | 18.7 | 64.8 | 20.1 (± 7.9) |
| CD8 Tte | 18.3 | 25.9 | 25.2 (± 11.3) |
| CD8 CD27+/CD28+ | 63.6 | 57.1 | 50.9 (± 15.2) |
| CD8 CD25+ | 0.7 | 1.7 | 1.1 (± 0.8) |

**A**

**(B)** Percentage of CD4/CD8 CAR+ subsets in CAR-T product, gated from total live T-cells. Percentage of Tn (CCR7+/CD45RA+), Tcm (CCR7+/CD45RA−), Te (CCR7−/CD45RA−) and Tte (CCR7−/CD45RA+) and CD27+/CD28+ subsets are sub gated from respective CD4/8 CAR+ populations. Key: PB = peripheral blood, Tn = naïve T-cells, Tcm = central memory T-cell, Te = effector T-cell and Tte = terminal T-cell.

| Product  Analysis (%) | CAR Product Analysis (%) | |
| --- | --- | --- |
|  | **Patient 1**  **(Cardiac)** | **Patient 2 (Renal)** |
| CD4 | 35.8 | 34 |
| CD8 | 22.6 | 51 |
| CD4 Tn | 1.7 | 0.3 |
| CD4 Tcm | 8.4 | 7.9 |
| CD4 Te | 88.5 | 91.9 |
| CD4 Tte | 1.5 | 0 |
| CD4 CD27+/CD28+ | 35.3 | 5.7 |
| CD8 Tn | 28 | 0.8 |
| CD8 Tcm | 1.5 | 1.2 |
| CD8 Te | 28 | 88.7 |
| CD8 Tte | 42.4 | 9.2 |
| CD8 CD27+/CD28+ | 13.6 | 0.6 |

**B**

**Sup. Table 4:** Longitudinal PB sampling post CAR-T infusion. Percentage CD4/CD8 CAR+ subsets in PB post CAR-T infusion, gated from total live cells. Percentage Tn (CCR7+/CD45RA+), Tcm (CCR7+/CD45RA−), Te (CCR7−/CD45RA−) and Tte (CCR7−/CD45RA+), CD27+/CD28+ and CD25+ subsets are sub gated from respective CD4/8 CAR+ populations. Percentage CAR-Tregs (CD127-/CD25+/FOXP3+) were combination gated from CD4 CAR+ populations. Key: PB = peripheral blood, Tn = naïve T-cells, Tcm = central memory T-cell, Te = effector T-cell and Tte = terminal T-cell.

| CAR Blood Analysis (%) | Day 7 | | Day 14 | | Day 23/28 | | Month 3 |
| --- | --- | --- | --- | --- | --- | --- | --- |
|  | **Patient 1**  **(Cardiac)** | **Patient 2**  **(Renal)** | **Patient 1**  **(Cardiac)** | **Patient 2**  **(Renal)** | **Patient 1**  **(Cardiac)** | **Patient 2**  **(Renal)** | **Patient 1**  **(Cardiac)** |
| CD4 | 1.8 | 5.8 | 0.7 | 3.2 | 3.8 | 2.5 | 0.4 |
| CD8 | 22.4 | 12.6 | 6.7 | 8.8 | 1.4 | 7.4 | 0.5 |
| CD4 Tn | 0.3 | 0.2 | 0.8 | 0 | 0.2 | 1.9 | 6.7 |
| CD4 Tcm | 30.8 | 5.6 | 24.1 | 4.6 | 19.2 | 5.7 | 40 |
| CD4 Te | 68.4 | 94.3 | 73.4 | 95.5 | 80.2 | 92.5 | 52.2 |
| CD4 Tte | 0.55 | 0 | 1.7 | 0 | 0.32 | 0 | 1.1 |
| CD4 CD27+/CD28+ | 82.9 | 48.4 | 75.6 | 27.3 | 37 | 58.5 | 91.1 |
| CD4 CD25+ | 30.2 | 68.2 | 31.3 | 100 | 31.9 | 65 | 33.3 |
| CD4 CD127-/CD25+/FOXP3+ | - | - | - | 66.7 | - | 52.2 | 25.6 |
| CD8 Tn | 1.7 | 0.1 | 1.2 | 0 | 6.3 | 0.6 | 12.8 |
| CD8 Tcm | 13.2 | 0.9 | 5.4 | 0 | 8.4 | 0.6 | 44 |
| CD8 Te | 75.6 | 74.8 | 65.8 | 66.7 | 70.9 | 66.9 | 31.2 |
| CD8 Tte | 9.5 | 24.2 | 27.5 | 33.3 | 14.4 | 31.9 | 11.9 |
| CD8 CD27+/CD28+ | 60.4 | 5.7 | 33.7 | 3.3 | 55.2 | 11.9 | 79.8 |
| CD8 CD25+ | 5.7 | 5.6 | 1.3 | 0 | 0.7 | 0 | 0.8 |

**SUP. FIGURE 1:**

**Sup. Figure 1.** **Imaging of patients with PTLD treated with commercial axi-cel.**

(A) PET imaging of P1 and P2 at Day -6 and at follow-up timepoints (Day 28, M3).

(B) P2: MRI brain coronal and sagittal views at Day+14 demonstrating lesions in the body of the right lateral ventricle and right splenium of the corpus callosum.


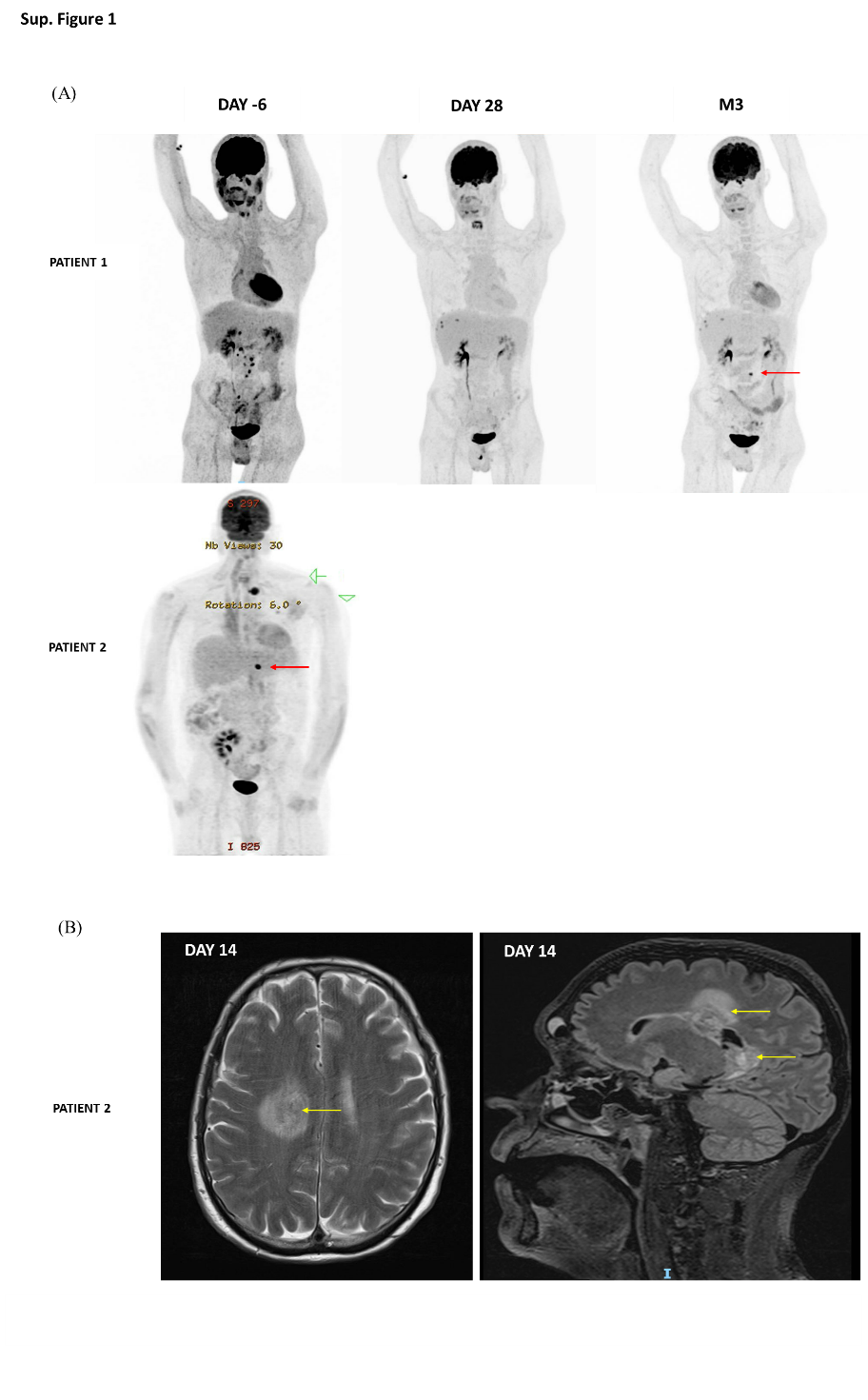


**REFERENCES:**

1. Swerdlow SH, Campo E, Pileri SA, Harris NL, Stein H, Siebert R, et al. The 2016 revision of the World Health Organization classification of lymphoid neoplasms. Blood. 2016 May 19;127(20):2375–90.

2. Lee DW, Santomasso BD, Locke FL, Ghobadi A, Turtle CJ, Brudno JN, et al. ASTCT Consensus Grading for Cytokine Release Syndrome and Neurologic Toxicity Associated with Immune Effector Cells. Biol Blood Marrow Transplant. 2019 Apr;25(4):625–38.

3. Cheson BD, Fisher RI, Barrington SF, Cavalli F, Schwartz LH, Zucca E, et al. Recommendations for Initial Evaluation, Staging, and Response Assessment of Hodgkin and Non-Hodgkin Lymphoma: The Lugano Classification. J Clin Oncol. 2014 Sep 20;32(27):3059–67.

4. Gattinoni L, Speiser DE, Lichterfeld M, Bonini C. T memory stem cells in health and disease. Nat Med. 2017 Jan;23(1):18–27.
